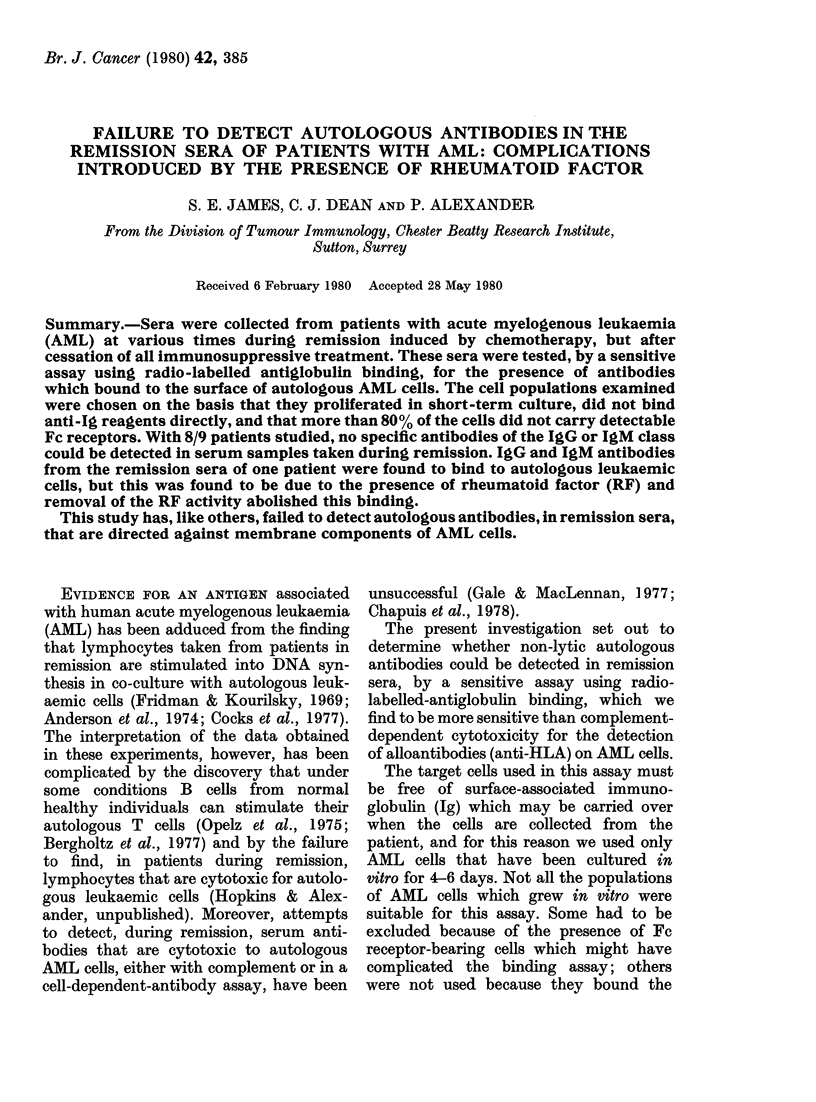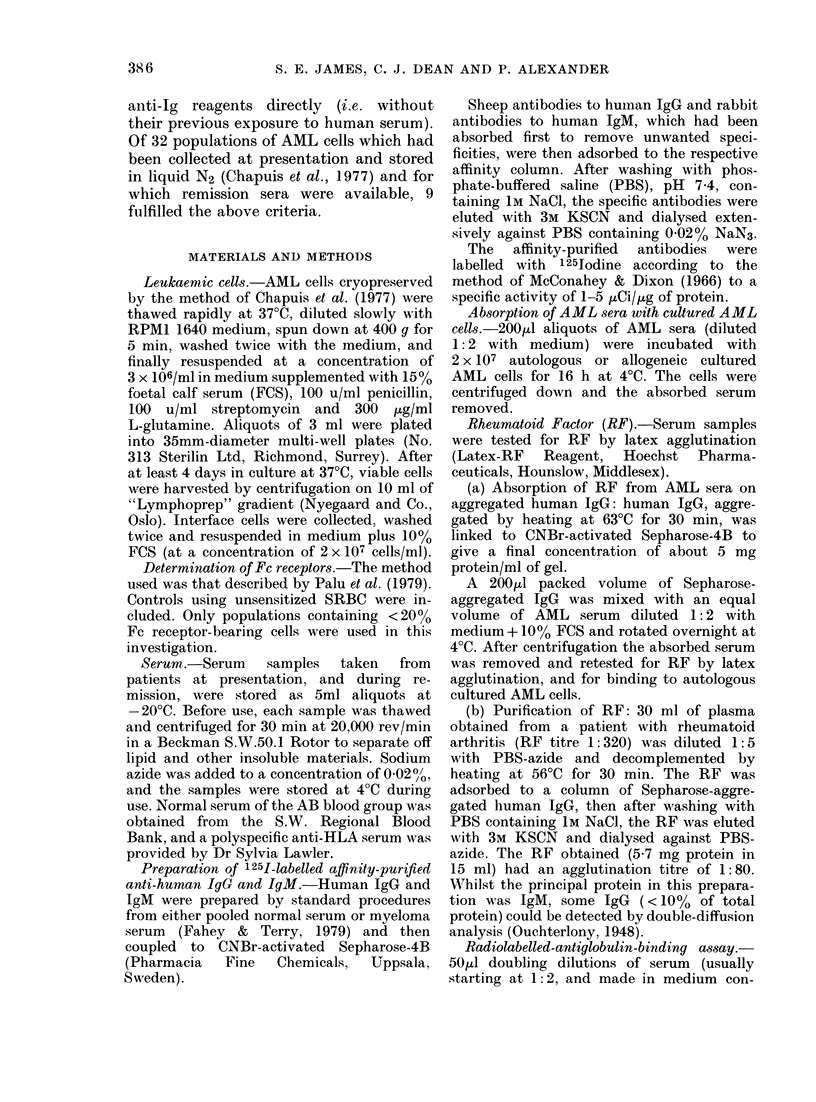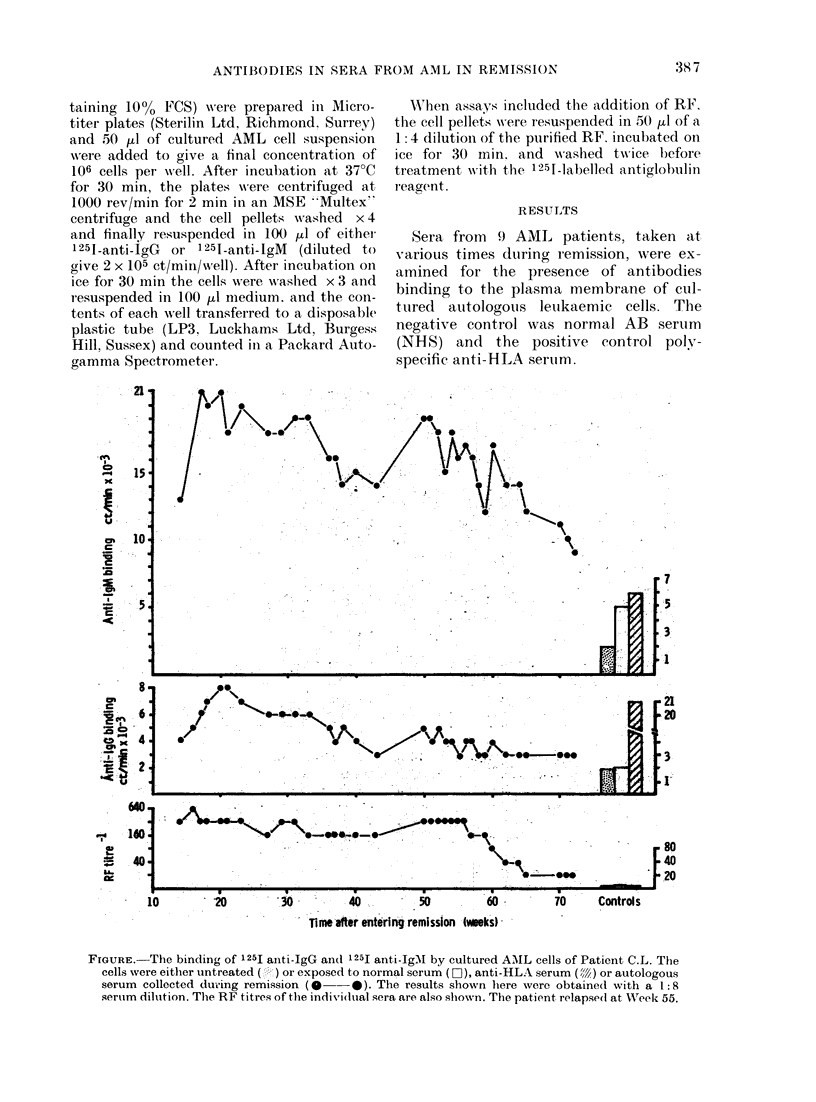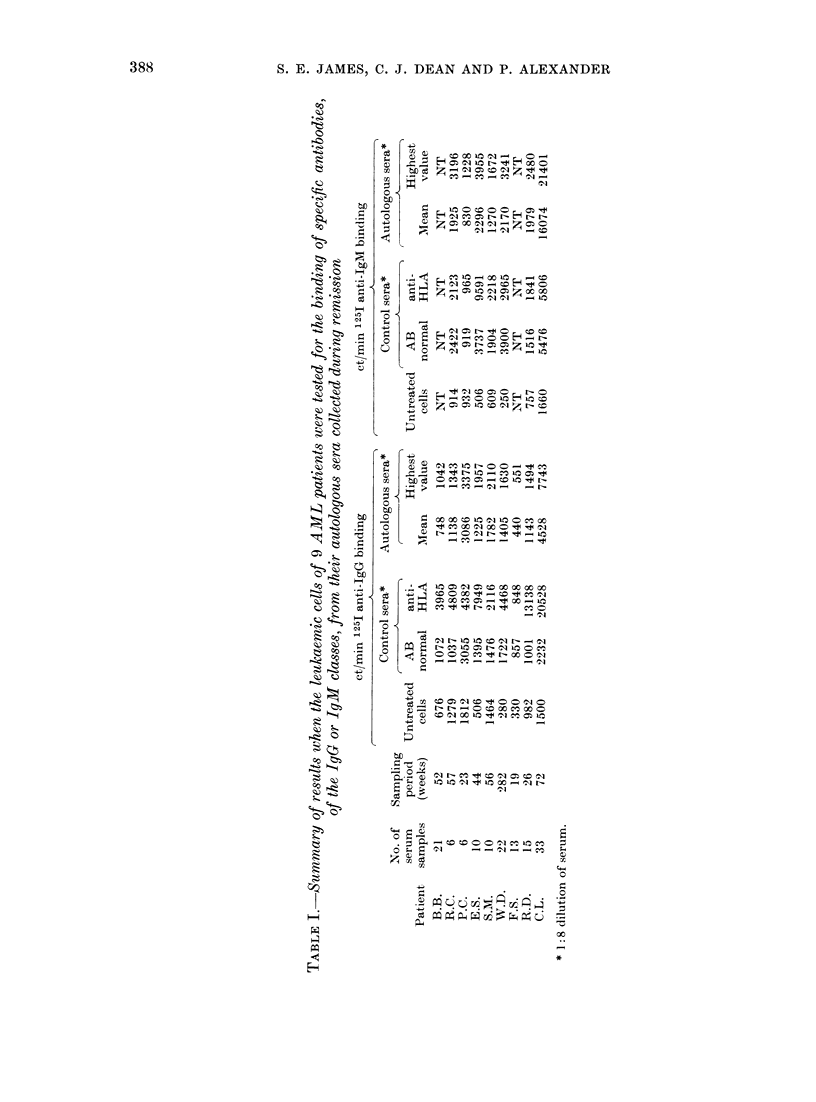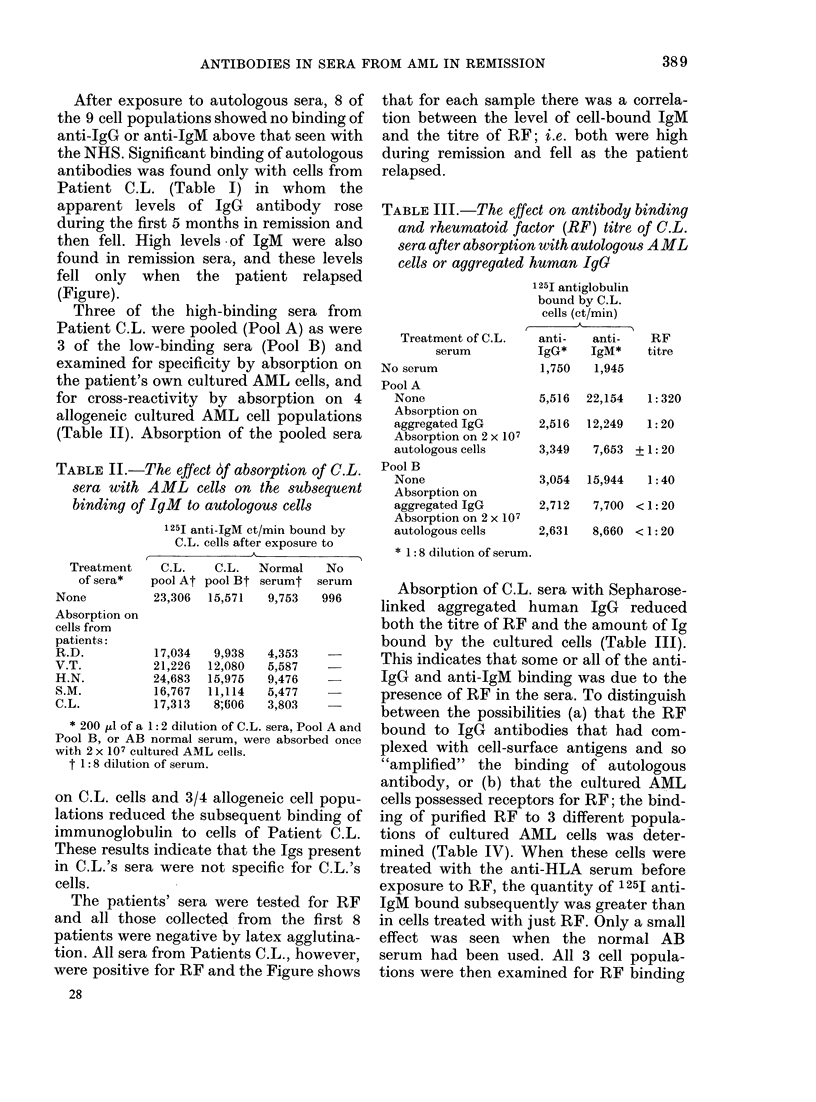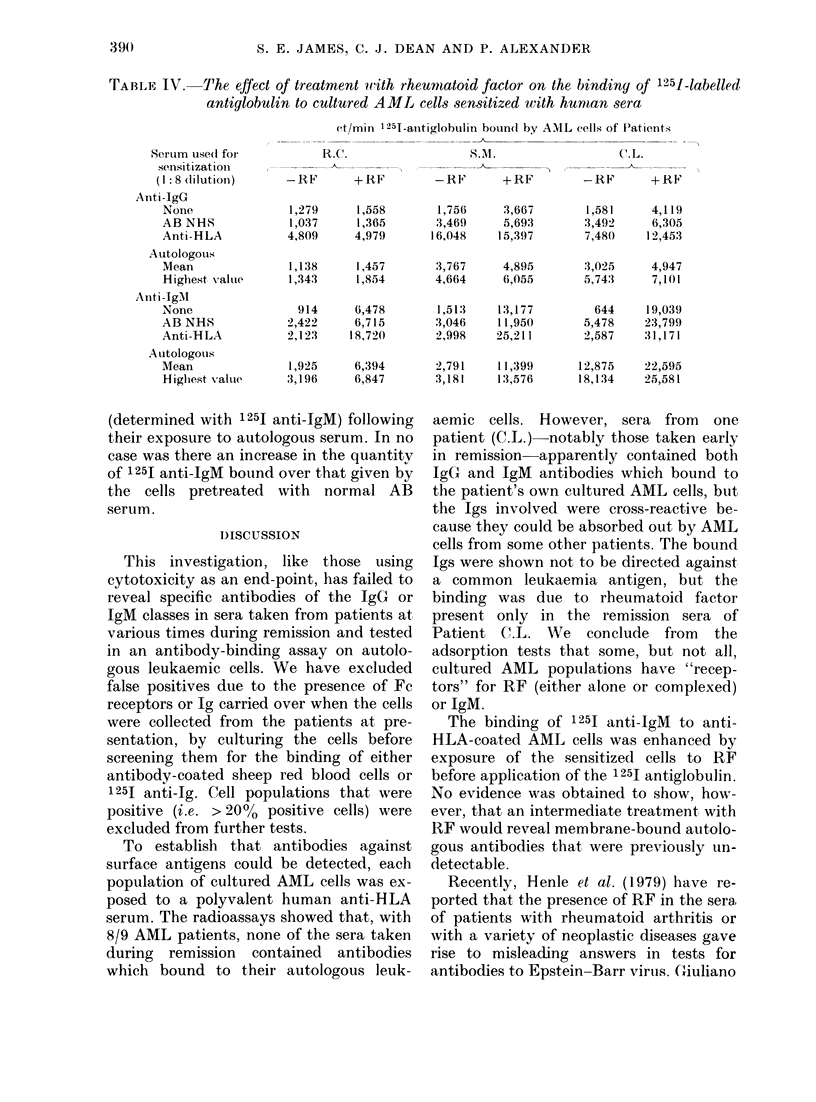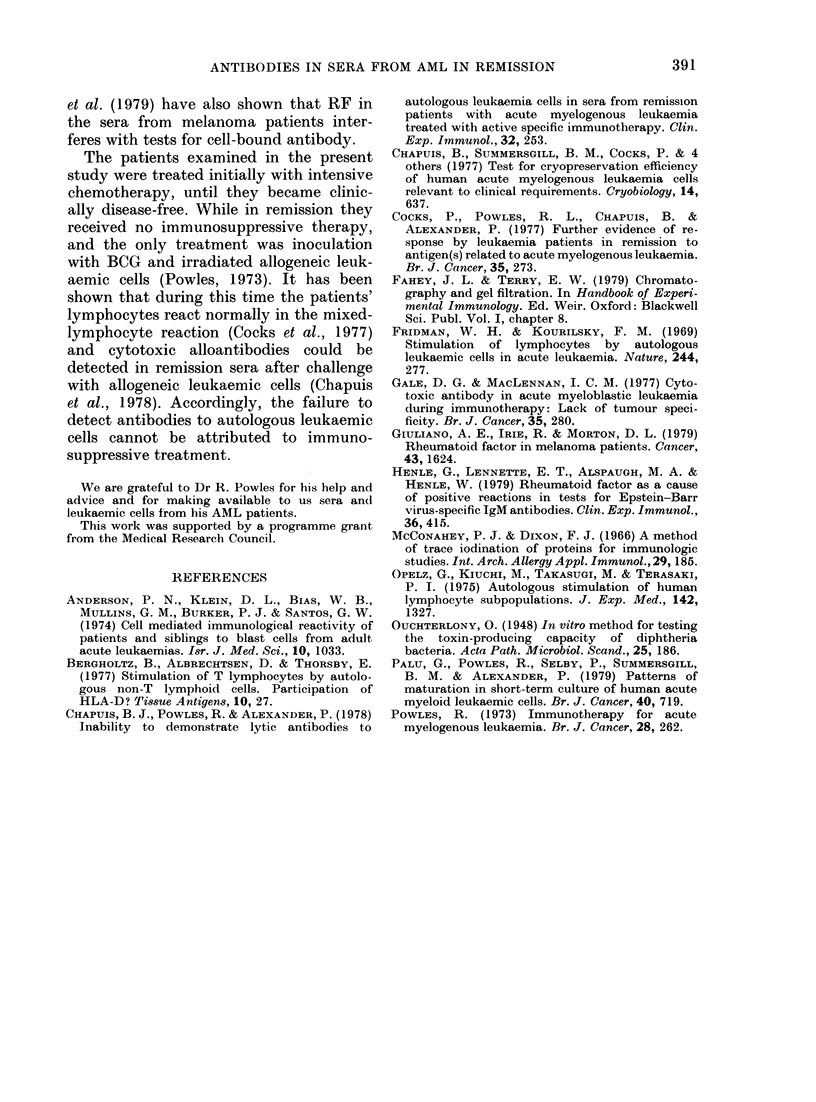# Failure to detect autologous antibodies in the remission sera of patients with AML: complications introduced by the presence of rheumatoid factor.

**DOI:** 10.1038/bjc.1980.249

**Published:** 1980-09

**Authors:** S. E. James, C. J. Dean, P. Alexander

## Abstract

Sera were collected from patients with acute myelogenous leukaemia (AML) at various times during remission induced by chemotherapy, but after cessation of all immunosupressive treatment. These sera were tested, by a sensitive assay using radio-labelled antiglobulin binding, for the presence of antibodies which bound to the surface of autologous AML cells. The cell populations examined were chosen on the basis that they proliferated in short-term culture, did not bind anti-Ig reagents directly, and that more than 80% of the cells did not carry detectable Fc receptors. With 8/9 patients studied, no specific antibodies of the IgG or IgM class could be detected in serum samples taken during remission. IgG and IgM antibodies from the remission sera of one patient were found be bind to autologous leukaemic cells, but this was found to be due to the presence of rheumatoid factor (RF) and removal of the RF activity abolished this binding. This study has, like others, failed to detect autologous antibodies, in remission sera, that are directed against membrane components of AML cells.


*Br. J. Cancer* (1980) **42**, 385

### FAILURE TO DETECT AUTOLOGOUS ANTIBODIES IN THE REMISSION SERA OF PATIENTS WITH AML: COMPLICATIONS INTRODUCED BY THE PRESENCE OF RHEUMATOID FACTOR

S. E. JAMES, C. J. DEAN AND P. ALEXANDER

*From the Division of Tumour Immunology, Chester Beatty Research Institute, Sutton, Surrey*

Received 6 February 1980 Accepted 28 May 1980

**Summary.—Sera were collected from patients with acute myelogenous leukaemia (AML) at various times during remission induced by chemotherapy, but after cessation of all immunosuppressive treatment. These sera were tested, by a sensitive assay using radio-labelled antiglobulin binding, for the presence of antibodies which bound to the surface of autologous AML cells. The cell populations examined were chosen on the basis that they proliferated in short-term culture, did not bind anti-Ig reagents directly, and that more than 80% of the cells did not carry detectable Fc receptors. With 8/9 patients studied, no specific antibodies of the IgG or IgM class could be detected in serum samples taken during remission. IgG and IgM antibodies from the remission sera of one patient were found to bind to autologous leukaemic cells, but this was found to be due to the presence of rheumatoid factor (RF) and removal of the RF activity abolished this binding.**

**This study has, like others, failed to detect autologous antibodies, in remission sera, that are directed against membrane components of AML cells.**

Evidence for an antigen associated with human acute myelogenous leukaemia (AML) has been adduced from the finding that lymphocytes taken from patients in remission are stimulated into DNA synthesis in co-culture with autologous leukaemic cells (Fridman & Kourilsky, 1969; Anderson *et al.*, 1974; Cocks *et al.*, 1977). The interpretation of the data obtained in these experiments, however, has been complicated by the discovery that under some conditions B cells from normal healthy individuals can stimulate their autologous T cells (Opelz *et al.*, 1975; Bergholtz *et al.*, 1977) and by the failure to find, in patients during remission, lymphocytes that are cytotoxic for autologous leukaemic cells (Hopkins & Alexander, unpublished). Moreover, attempts to detect, during remission, serum antibodies that are cytotoxic to autologous AML cells, either with complement or in a cell-dependent-antibody assay, have been unsuccessful (Gale & MacLennan, 1977; Chapuis *et al.*, 1978).

The present investigation set out to determine whether non-lytic autologous antibodies could be detected in remission sera, by a sensitive assay using radio-labelled-antiglobulin binding, which we find to be more sensitive than complement-dependent cytotoxicity for the detection of alloantibodies (anti-HLA) on AML cells.

The target cells used in this assay must be free of surface-associated immunoglobulin (Ig) which may be carried over when the cells are collected from the patient, and for this reason we used only AML cells that have been cultured *in vitro* for 4–6 days. Not all the populations of AML cells which grew *in vitro* were suitable for this assay. Some had to be excluded because of the presence of Fc receptor-bearing cells which might have complicated the binding assay; others were not used because they bound the anti-Ig reagents directly (*i.e.* without their previous exposure to human serum). Of 32 populations of AML cells which had been collected at presentation and stored in liquid $N_2$ (Chapuis *et al.*, 1977) and for which remission sera were available, 9 fulfilled the above criteria.

### MATERIALS AND METHODS

*Leukaemic cells.*—AML cells cryopreserved by the method of Chapuis *et al.* (1977) were thawed rapidly at 37°C, diluted slowly with RPM1 1640 medium, spun down at 400 *g* for 5 min, washed twice with the medium, and finally resuspended at a concentration of $3 \times 10^6$/ml in medium supplemented with 15% foetal calf serum (FCS), 100 u/ml penicillin, 100 u/ml streptomycin and 300 μg/ml L-glutamine. Aliquots of 3 ml were plated into 35mm-diameter multi-well plates (No. 313 Sterilin Ltd, Richmond, Surrey). After at least 4 days in culture at 37°C, viable cells were harvested by centrifugation on 10 ml of "Lymphoprep" gradient (Nyegaard and Co., Oslo). Interface cells were collected, washed twice and resuspended in medium plus 10% FCS (at a concentration of $2 \times 10^7$ cells/ml).

*Determination of Fc receptors.*—The method used was that described by Palu *et al.* (1979). Controls using unsensitized SRBC were included. Only populations containing <20% Fc receptor-bearing cells were used in this investigation.

*Serum.*—Serum samples taken from patients at presentation, and during remission, were stored as 5ml aliquots at −20°C. Before use, each sample was thawed and centrifuged for 30 min at 20,000 rev/min in a Beckman S.W.50.1 Rotor to separate off lipid and other insoluble materials. Sodium azide was added to a concentration of 0·02%, and the samples were stored at 4°C during use. Normal serum of the AB blood group was obtained from the S.W. Regional Blood Bank, and a polyspecific anti-HLA serum was provided by Dr Sylvia Lawler.

*Preparation of $^{125}$I-labelled affinity-purified anti-human IgG and IgM.*—Human IgG and IgM were prepared by standard procedures from either pooled normal serum or myeloma serum (Fahey & Terry, 1979) and then coupled to CNBr-activated Sepharose-4B (Pharmacia Fine Chemicals, Uppsala, Sweden).

Sheep antibodies to human IgG and rabbit antibodies to human IgM, which had been absorbed first to remove unwanted specificities, were then adsorbed to the respective affinity column. After washing with phosphate-buffered saline (PBS), pH 7·4, containing 1M NaCl, the specific antibodies were eluted with 3M KSCN and dialysed extensively against PBS containing 0·02% $NaN_3$.

The affinity-purified antibodies were labelled with $^{125}$Iodine according to the method of McConahey & Dixon (1966) to a specific activity of 1–5 μCi/μg of protein.

*Absorption of AML sera with cultured AML cells.*—200μl aliquots of AML sera (diluted 1:2 with medium) were incubated with $2 \times 10^7$ autologous or allogeneic cultured AML cells for 16 h at 4°C. The cells were centrifuged down and the absorbed serum removed.

*Rheumatoid Factor (RF).*—Serum samples were tested for RF by latex agglutination (Latex-RF Reagent, Hoechst Pharmaceuticals, Hounslow, Middlesex).

(a) Absorption of RF from AML sera on aggregated human IgG: human IgG, aggregated by heating at 63°C for 30 min, was linked to CNBr-activated Sepharose-4B to give a final concentration of about 5 mg protein/ml of gel.

A 200μl packed volume of Sepharose-aggregated IgG was mixed with an equal volume of AML serum diluted 1:2 with medium + 10% FCS and rotated overnight at 4°C. After centrifugation the absorbed serum was removed and retested for RF by latex agglutination, and for binding to autologous cultured AML cells.

(b) Purification of RF: 30 ml of plasma obtained from a patient with rheumatoid arthritis (RF titre 1:320) was diluted 1:5 with PBS-azide and decomplemented by heating at 56°C for 30 min. The RF was adsorbed to a column of Sepharose-aggregated human IgG, then after washing with PBS containing 1M NaCl, the RF was eluted with 3M KSCN and dialysed against PBS-azide. The RF obtained (5·7 mg protein in 15 ml) had an agglutination titre of 1:80. Whilst the principal protein in this preparation was IgM, some IgG (<10% of total protein) could be detected by double-diffusion analysis (Ouchterlony, 1948).

*Radiolabelled-antiglobulin-binding assay.*—50μl doubling dilutions of serum (usually starting at 1:2, and made in medium containing 10% FCS) were prepared in Microtiter plates (Sterilin Ltd, Richmond, Surrey) and 50 $\mu$l of cultured AML cell suspension were added to give a final concentration of $10^6$ cells per well. After incubation at 37°C for 30 min, the plates were centrifuged at 1000 rev/min for 2 min in an MSE "Multex" centrifuge and the cell pellets washed $\times 4$ and finally resuspended in 100 $\mu$l of either $^{125}$I-anti-IgG or $^{125}$I-anti-IgM (diluted to give $2 \times 10^5$ ct/min/well). After incubation on ice for 30 min the cells were washed $\times 3$ and resuspended in 100 $\mu$l medium, and the contents of each well transferred to a disposable plastic tube (LP3, Luckhams Ltd, Burgess Hill, Sussex) and counted in a Packard Autogamma Spectrometer.

When assays included the addition of RF, the cell pellets were resuspended in 50 $\mu$l of a 1:4 dilution of the purified RF, incubated on ice for 30 min, and washed twice before treatment with the $^{125}$I-labelled antiglobulin reagent.

### RESULTS

Sera from 9 AML patients, taken at various times during remission, were examined for the presence of antibodies binding to the plasma membrane of cultured autologous leukaemic cells. The negative control was normal AB serum (NHS) and the positive control polyspecific anti-HLA serum.

FIGURE.—The binding of $^{125}$I anti-IgG and $^{125}$I anti-IgM by cultured AML cells of Patient C.L. The cells were either untreated (░) or exposed to normal serum (□), anti-HLA serum (▨) or autologous serum collected during remission (●——●). The results shown here were obtained with a 1:8 serum dilution. The RF titres of the individual sera are also shown. The patient relapsed at Week 55.

TABLE I.—*Summary of results when the leukaemic cells of 9 AML patients were tested for the binding of specific antibodies, of the IgG or IgM classes, from their autologous sera collected during remission*

| | | | ct/min $^{125}$I anti-IgG binding | | | | | ct/min $^{125}$I anti-IgM binding | | | | |
|---|---|---|---|---|---|---|---|---|---|---|---|---|
| | | | | Control sera* | | Autologous sera* | | | Control sera* | | Autologous sera* | |
| Patient | No. of serum samples | Sampling period (weeks) | Untreated cells | AB normal | anti-HLA | Mean | Highest value | Untreated cells | AB normal | anti-HLA | Mean | Highest value |
| B.B. | 21 | 52 | 676 | 1072 | 3965 | 748 | 1042 | NT | NT | NT | NT | NT |
| R.C. | 6 | 57 | 1279 | 1037 | 4809 | 1138 | 1343 | 914 | 2422 | 2123 | 1925 | 3196 |
| P.C. | 6 | 23 | 1812 | 3055 | 4382 | 3086 | 3375 | 932 | 919 | 965 | 830 | 1228 |
| E.S. | 10 | 44 | 506 | 1395 | 7949 | 1225 | 1957 | 506 | 3737 | 9591 | 2296 | 3955 |
| S.M. | 10 | 56 | 1464 | 1476 | 2116 | 1782 | 2110 | 609 | 1904 | 2218 | 1270 | 1672 |
| W.D. | 22 | 282 | 280 | 1722 | 4468 | 1405 | 1630 | 250 | 3900 | 2965 | 2170 | 3241 |
| F.S. | 13 | 19 | 330 | 857 | 848 | 440 | 551 | NT | NT | NT | NT | NT |
| R.D. | 15 | 26 | 982 | 1001 | 13138 | 1143 | 1494 | 757 | 1516 | 1841 | 1979 | 2480 |
| C.L. | 33 | 72 | 1500 | 2232 | 20528 | 4528 | 7743 | 1660 | 5476 | 5806 | 16074 | 21401 |

* 1:8 dilution of serum.

After exposure to autologous sera, 8 of the 9 cell populations showed no binding of anti-IgG or anti-IgM above that seen with the NHS. Significant binding of autologous antibodies was found only with cells from Patient C.L. (Table I) in whom the apparent levels of IgG antibody rose during the first 5 months in remission and then fell. High levels of IgM were also found in remission sera, and these levels fell only when the patient relapsed (Figure).

Three of the high-binding sera from Patient C.L. were pooled (Pool A) as were 3 of the low-binding sera (Pool B) and examined for specificity by absorption on the patient's own cultured AML cells, and for cross-reactivity by absorption on 4 allogeneic cultured AML cell populations (Table II). Absorption of the pooled sera on C.L. cells and 3/4 allogeneic cell populations reduced the subsequent binding of immunoglobulin to cells of Patient C.L. These results indicate that the Igs present in C.L.'s sera were not specific for C.L.'s cells.

TABLE II.—*The effect of absorption of C.L. sera with AML cells on the subsequent binding of IgM to autologous cells*

| Treatment of sera* | $^{125}$I anti-IgM ct/min bound by C.L. cells after exposure to: C.L. pool A† | C.L. pool B† | Normal serum† | No serum |
|---|---|---|---|---|
| None | 23,306 | 15,571 | 9,753 | 996 |
| Absorption on cells from patients: | | | | |
| R.D. | 17,034 | 9,938 | 4,353 | — |
| V.T. | 21,226 | 12,080 | 5,587 | — |
| H.N. | 24,683 | 15,975 | 9,476 | — |
| S.M. | 16,767 | 11,114 | 5,477 | — |
| C.L. | 17,313 | 8,606 | 3,803 | — |

* 200 μl of a 1:2 dilution of C.L. sera, Pool A and Pool B, or AB normal serum, were absorbed once with $2 \times 10^7$ cultured AML cells.
† 1:8 dilution of serum.

The patients' sera were tested for RF and all those collected from the first 8 patients were negative by latex agglutination. All sera from Patients C.L., however, were positive for RF and the Figure shows that for each sample there was a correlation between the level of cell-bound IgM and the titre of RF; *i.e.* both were high during remission and fell as the patient relapsed.

TABLE III.—*The effect on antibody binding and rheumatoid factor (RF) titre of C.L. sera after absorption with autologous AML cells or aggregated human IgG*

| Treatment of C.L. serum | $^{125}$I antiglobulin bound by C.L. cells (ct/min): anti-IgG* | anti-IgM* | RF titre |
|---|---|---|---|
| No serum | 1,750 | 1,945 | |
| Pool A | | | |
| None | 5,516 | 22,154 | 1:320 |
| Absorption on aggregated IgG | 2,516 | 12,249 | 1:20 |
| Absorption on $2 \times 10^7$ autologous cells | 3,349 | 7,653 | ±1:20 |
| Pool B | | | |
| None | 3,054 | 15,944 | 1:40 |
| Absorption on aggregated IgG | 2,712 | 7,700 | <1:20 |
| Absorption on $2 \times 10^7$ autologous cells | 2,631 | 8,660 | <1:20 |

* 1:8 dilution of serum.

Absorption of C.L. sera with Sepharose-linked aggregated human IgG reduced both the titre of RF and the amount of Ig bound by the cultured cells (Table III). This indicates that some or all of the anti-IgG and anti-IgM binding was due to the presence of RF in the sera. To distinguish between the possibilities (a) that the RF bound to IgG antibodies that had complexed with cell-surface antigens and so "amplified" the binding of autologous antibody, or (b) that the cultured AML cells possessed receptors for RF; the binding of purified RF to 3 different populations of cultured AML cells was determined (Table IV). When these cells were treated with the anti-HLA serum before exposure to RF, the quantity of $^{125}$I anti-IgM bound subsequently was greater than in cells treated with just RF. Only a small effect was seen when the normal AB serum had been used. All 3 cell populations were then examined for RF binding

TABLE IV.—*The effect of treatment with rheumatoid factor on the binding of $^{125}$I-labelled antiglobulin to cultured AML cells sensitized with human sera*

| Serum used for sensitization (1:8 dilution) | ct/min $^{125}$I-antiglobulin bound by AML cells of Patients: R.C. −RF | R.C. +RF | S.M. −RF | S.M. +RF | C.L. −RF | C.L. +RF |
|---|---|---|---|---|---|---|
| Anti-IgG | | | | | | |
| None | 1,279 | 1,558 | 1,756 | 3,667 | 1,581 | 4,119 |
| AB NHS | 1,037 | 1,365 | 3,469 | 5,693 | 3,492 | 6,305 |
| Anti-HLA | 4,809 | 4,979 | 16,048 | 15,397 | 7,480 | 12,453 |
| Autologous | | | | | | |
| Mean | 1,138 | 1,457 | 3,767 | 4,895 | 3,025 | 4,947 |
| Highest value | 1,343 | 1,854 | 4,664 | 6,055 | 5,743 | 7,101 |
| Anti-IgM | | | | | | |
| None | 914 | 6,478 | 1,513 | 13,177 | 644 | 19,039 |
| AB NHS | 2,422 | 6,715 | 3,046 | 11,950 | 5,478 | 23,799 |
| Anti-HLA | 2,123 | 18,720 | 2,998 | 25,211 | 2,587 | 31,171 |
| Autologous | | | | | | |
| Mean | 1,925 | 6,394 | 2,791 | 11,399 | 12,875 | 22,595 |
| Highest value | 3,196 | 6,847 | 3,181 | 13,576 | 18,134 | 25,581 |

(determined with $^{125}$I anti-IgM) following their exposure to autologous serum. In no case was there an increase in the quantity of $^{125}$I anti-IgM bound over that given by the cells pretreated with normal AB serum.

### DISCUSSION

This investigation, like those using cytotoxicity as an end-point, has failed to reveal specific antibodies of the IgG or IgM classes in sera taken from patients at various times during remission and tested in an antibody-binding assay on autologous leukaemic cells. We have excluded false positives due to the presence of Fc receptors or Ig carried over when the cells were collected from the patients at presentation, by culturing the cells before screening them for the binding of either antibody-coated sheep red blood cells or $^{125}$I anti-Ig. Cell populations that were positive (*i.e.* $>20\%$ positive cells) were excluded from further tests.

To establish that antibodies against surface antigens could be detected, each population of cultured AML cells was exposed to a polyvalent human anti-HLA serum. The radioassays showed that, with 8/9 AML patients, none of the sera taken during remission contained antibodies which bound to their autologous leukaemic cells. However, sera from one patient (C.L.)—notably those taken early in remission—apparently contained both IgG and IgM antibodies which bound to the patient's own cultured AML cells, but the Igs involved were cross-reactive because they could be absorbed out by AML cells from some other patients. The bound Igs were shown not to be directed against a common leukaemia antigen, but the binding was due to rheumatoid factor present only in the remission sera of Patient C.L. We conclude from the adsorption tests that some, but not all, cultured AML populations have "receptors" for RF (either alone or complexed) or IgM.

The binding of $^{125}$I anti-IgM to anti-HLA-coated AML cells was enhanced by exposure of the sensitized cells to RF before application of the $^{125}$I antiglobulin. No evidence was obtained to show, however, that an intermediate treatment with RF would reveal membrane-bound autologous antibodies that were previously undetectable.

Recently, Henle *et al.* (1979) have reported that the presence of RF in the sera of patients with rheumatoid arthritis or with a variety of neoplastic diseases gave rise to misleading answers in tests for antibodies to Epstein–Barr virus. Giuliano

*et al.* (1979) have also shown that RF in the sera from melanoma patients interferes with tests for cell-bound antibody.

The patients examined in the present study were treated initially with intensive chemotherapy, until they became clinically disease-free. While in remission they received no immunosuppressive therapy, and the only treatment was inoculation with BCG and irradiated allogeneic leukaemic cells (Powles, 1973). It has been shown that during this time the patients' lymphocytes react normally in the mixed-lymphocyte reaction (Cocks *et al.*, 1977) and cytotoxic alloantibodies could be detected in remission sera after challenge with allogeneic leukaemic cells (Chapuis *et al.*, 1978). Accordingly, the failure to detect antibodies to autologous leukaemic cells cannot be attributed to immunosuppressive treatment.

We are grateful to Dr R. Powles for his help and advice and for making available to us sera and leukaemic cells from his AML patients.

This work was supported by a programme grant from the Medical Research Council.

### REFERENCES

Anderson, P. N., Klein, D. L., Bias, W. B., Mullins, G. M., Burker, P. J. & Santos, G. W. (1974) Cell mediated immunological reactivity of patients and siblings to blast cells from adult acute leukaemias. *Isr. J. Med. Sci.*, **10**, 1033.

Bergholtz, B., Albrechtsen, D. & Thorsby, E. (1977) Stimulation of T lymphocytes by autologous non-T lymphoid cells. Participation of HLA-D? *Tissue Antigens*, **10**, 27.

Chapuis, B. J., Powles, R. & Alexander, P. (1978) Inability to demonstrate lytic antibodies to autologous leukaemia cells in sera from remission patients with acute myelogenous leukaemia treated with active specific immunotherapy. *Clin. Exp. Immunol.*, **32**, 253.

Chapuis, B., Summersgill, B. M., Cocks, P. & 4 others (1977) Test for cryopreservation efficiency of human acute myelogenous leukaemia cells relevant to clinical requirements. *Cryobiology*, **14**, 637.

Cocks, P., Powles, R. L., Chapuis, B. & Alexander, P. (1977) Further evidence of response by leukaemia patients in remission to antigen(s) related to acute myelogenous leukaemia. *Br. J. Cancer*, **35**, 273.

Fahey, J. L. & Terry, E. W. (1979) Chromatography and gel filtration. In *Handbook of Experimental Immunology*. Ed. Weir. Oxford: Blackwell Sci. Publ. Vol. I, chapter 8.

Fridman, W. H. & Kourilsky, F. M. (1969) Stimulation of lymphocytes by autologous leukaemic cells in acute leukaemia. *Nature*, **244**, 277.

Gale, D. G. & MacLennan, I. C. M. (1977) Cytotoxic antibody in acute myeloblastic leukaemia during immunotherapy: Lack of tumour specificity. *Br. J. Cancer*, **35**, 280.

Giuliano, A. E., Irie, R. & Morton, D. L. (1979) Rheumatoid factor in melanoma patients. *Cancer*, **43**, 1624.

Henle, G., Lennette, E. T., Alspaugh, M. A. & Henle, W. (1979) Rheumatoid factor as a cause of positive reactions in tests for Epstein–Barr virus-specific IgM antibodies. *Clin. Exp. Immunol.*, **36**, 415.

McConahey, P. J. & Dixon, F. J. (1966) A method of trace iodination of proteins for immunologic studies. *Int. Arch. Allergy Appl. Immunol.*, **29**, 185.

Opelz, G., Kiuchi, M., Takasugi, M. & Terasaki, P. I. (1975) Autologous stimulation of human lymphocyte subpopulations. *J. Exp. Med.*, **142**, 1327.

Ouchterlony, O. (1948) *In vitro* method for testing the toxin-producing capacity of diphtheria bacteria. *Acta Path. Microbiol. Scand.*, **25**, 186.

Palu, G., Powles, R., Selby, P., Summersgill, B. M. & Alexander, P. (1979) Patterns of maturation in short-term culture of human acute myeloid leukaemic cells. *Br. J. Cancer*, **40**, 719.

Powles, R. (1973) Immunotherapy for acute myelogenous leukaemia. *Br. J. Cancer*, **28**, 262.